# Implementing corrections of isocentric shifts for the stereotactic irradiation of cerebral targets: Clinical validation

**DOI:** 10.1002/acm2.13577

**Published:** 2022-03-02

**Authors:** Sonja Wegener, Robert Schindhelm, Otto A. Sauer

**Affiliations:** ^1^ Department of Radiation Oncology University of Wuerzburg Wuerzburg Germany

**Keywords:** isocenter, quality assurance, stereotactic irradiation

## Abstract

**Purpose**: Any Linac will show geometric imprecisions, including non‐ideal alignment of the gantry, collimator and couch axes, and gantry sag or wobble. Their angular dependence can be quantified and resulting changes of the dose distribution predicted (Wack, JACMP 20(5), 2020). We analyzed whether it is feasible to correct geometric shifts during treatment planning. The successful implementation of such a correction procedure was verified by measurements of different stereotactic treatment plans.

**Methods**: Isocentric shifts were quantified for two Elekta Synergy Agility Linacs using the QualiForMed ISO‐CBCT+ module, yielding the shift between kV and MV isocenters, the gantry flex and wobble as well as the positions of couch and collimator rotation axes. Next, the position of each field's isocenter in the Pinnacle treatment planning system was adjusted accordingly using a script. Fifteen stereotactic treatment plans of cerebral metastases (0.34 to 26.53 cm^3^) comprising 9–11 beams were investigated; 54 gantry and couch combinations in total. Unmodified plans and corrected plans were measured using the Sun Nuclear SRS‐MapCHECK with the Stereophan phantom and evaluated using gamma analysis.

**Results**: Geometric imprecisions, such as shifts of up to 0.8 mm between kV and MV isocenter, a couch rotation axis 0.9 mm off the kV isocente,r and gantry flex with an amplitude of 1.1 mm, were found. For eight, mostly small PTVs D98 values declined more than 5% by simulating these shifts. The average gamma (2%/2 mm, absolute, global, 20% threshold) was reduced from 0.53 to 0.31 (0.32 to 0.30) for Linac 1 (Linac 2) when including the isocentric corrections. Thus, Linac 1 reached the accuracy level of Linac 2 after correction.

**Conclusion**: Correcting for Linac geometric deviations during the planning process is feasible and was dosimetrically validated. The dosimetric impact of the geometric imperfections can vary between Linacs and should be assessed and corrected where necessary.

## 1 INTRODUCTION

Irradiation of small intracranial lesions requires high accuracy. Several recommendations exist for technical requirements for the geometric accuracy of stereotactic treatment machines, the more stringent demanding 1 mm localization accuracy in end‐to‐end tests^1^, ^2^ or 5%^1^ to 3%^2s^ dose agreement between measured and calculated dose. While dedicated machines for high precision and accuracy irradiations exist, these recommendations can typically also be met with conventional linear accelerators (Linacs). Despite all efforts to improve patient positioning and delivery accuracy, mechanical imperfections of these machines remain.^3^, ^4^, ^5^, ^6^, ^7^, ^8^ With the increasing consideration of irradiation of multiple metastases as a treatment option,^9^ and the increasing use of single‐isocenter multi‐target irradiations,^10^ geometric problems come even more into the focus.

These include the alignment of the different Linac rotation axes (gantry, collimator, couch), the positioning of MLC banks and individual leaves, the alignment of the MV isocenter with the kV imaging isocenter, gravitational effects such as gantry sagging and mechanical play of different components. Machine quality assurance typically covers these aspects. The Linac isocenter position is often evaluated by star shots on film, 3D radiochromic dosimeters,^3^ or by using the Winston‐Lutz test^11^ in combination with the portal imager. All tests are, however, limited to spatial verification excluding dosimetric information. The dosimetric verification of the irradiation accuracy on a plan‐by‐plan basis can be performed using dedicated measurement devices.^12^, ^13^

Different geometric deviations affect a dose distribution by different degrees. Wack et al.^4^ analyzed the impact of different geometric imperfections on small circular cerebral lesions in a planning study, finding that the dose to the target volume can change to a clinically relevant degree due to realistic offsets between MV and kV isocenters, missing alignment of the rotation axes and due to gantry sag. It was further shown that a measured dose distribution was more similar to a plan in which the anticipated geometric shifts were included than to the original plan. This validated the implementation of the proposed correction. The suggested procedure did predict the changes, but did not correct for the geometric deviations before treatment.

These findings need inclusion into the clinical planning process to improve the irradiation accuracy. Consequently, a different planning protocol is necessary and plan‐by‐plan validation seems necessary. This work reports on the procedure used to include the necessary corrections into the treatment planning process and its application to fifteen different clinical stereotactic treatment plans for brain lesions covering a wide range of different angular combinations and irradiation volumes on two different Linacs. The successful application of the correction procedure was validated by measurements.

## 2 MATERIALS AND METHODS

### 2.1 Quantification of isocenter shifts

Isocentric shifts were quantified for two Elekta Synergy Linacs with Agility MLC (Elekta Medical Systems, Crawley, UK) using Winston–Lutz tests. In more detail, the patented test object called OTP‐ISO+ (QualiForMed, La Roche Sur Yon, France) was used. The OTP‐ISO+ test object is a plastic sphere containing several metallic balls including a central one with 5 mm diameter. The metallic balls are visible in the taken images and provide information about the position of the isocenter using the central ball and on the gantry angle using the outer balls. The test object was aligned to the lasers in a preparatory step. kV CBCT imaging was performed in a full clockwise rotation using the collimator and filter cassettes combination labeled F0S10 (F0: blank filter with no effect on the X‐ray beam, S10: cassette with an opening that creates a small field of view with a diameter of 250 mm and a nominal irradiated length at isocenter of 135.4 mm). The shift between the initial position and the isocenter of the kV image was determined using the Linac's XVI software. The phantom was moved by the indicated amount using the iGuide software (Medical Intelligence, Schwabmuenchen, version 2.2.3) and a robotic Hexapod couch. The new position was again verified with a second CBCT using the same preset but the opposite rotation direction. This procedure tightly adheres to our clinical protocol for stereotactic treatments. It also means that the obtained corrections later used in the correction script are valid only for positioning that is carried out using CBCT imaging as all indicated distances refer to the kV or MV isocenter. The laser position is irrelevant for the correction script.

To test the alignment of the MV isocenter and the test object at its position in the kV isocenter, MV images of 15 × 15 cm^2^ sized fields at 6 MV were acquired at 20 different gantry angles and 20 different collimator angles, both spaced across 360°, and 13 different couch angles ranging from 90° to 270°.

Analysis was performed using QualiForMed Qualimagic (version 6.17.5) software module ISO‐CBCT+, which is based on the work of Winkler et al.^14^ The offset vector between kV and MV isocenter as well as the offsets between couch or collimator rotation axes and the gantry rotation axis, gantry flex, and wobble were obtained from the software. In more detail, the Linac MV isocenter was defined as the point with the smallest distance to all three rotation axes (gantry, collimator, and couch). The position of each axis and the kV imaging center with respect to the Linac isocenter was detailed in the analysis protocol together with graphical representations of gantry sag and wobble. Here, sag refers to the movement of the central beam along the gun‐target axis during gantry rotation, while wobble refers to movements in the plane perpendicular to that axis. For the full numerical information of the gantry sag and wobble, the coordinates of the central ball as well as the positions of the field edges along all directions were retrieved from the software's step‐by‐step mode. From these coordinates, the gantry angle‐dependent shifts were calculated.

The measurements were repeated 8 times varying the collimator angle during the gantry rotation from 0° to 90°, 180°, and 270° to account for any collimator angle‐dependent effect as well as using a gantry rotation in a clockwise and counter‐clockwise direction to assess its effect on sag and wobble. The results were averaged to obtain the values applied in the correction.

### 2.2 Correction procedure

The direction of the beam is slightly shifted with a gantry angle, which leads to a gantry‐dependent isocenter. The target point slightly moves with couch angle, which adds to the isocentric shift. Combining the corrections is straight forward. A script generated using Pinnacle (Philips Radiation Oncology Systems, Fitchburg, WI, version 16.2) presented by Wack et al.^4^ creates the true isocenter per beam direction. This is the nominal isocenter shifted due to gantry sag and wobble, the distance between CBCT and MV isocenter, the distance of the collimator axis and couch axis to the gantry axis.

**FIGURE 1:**
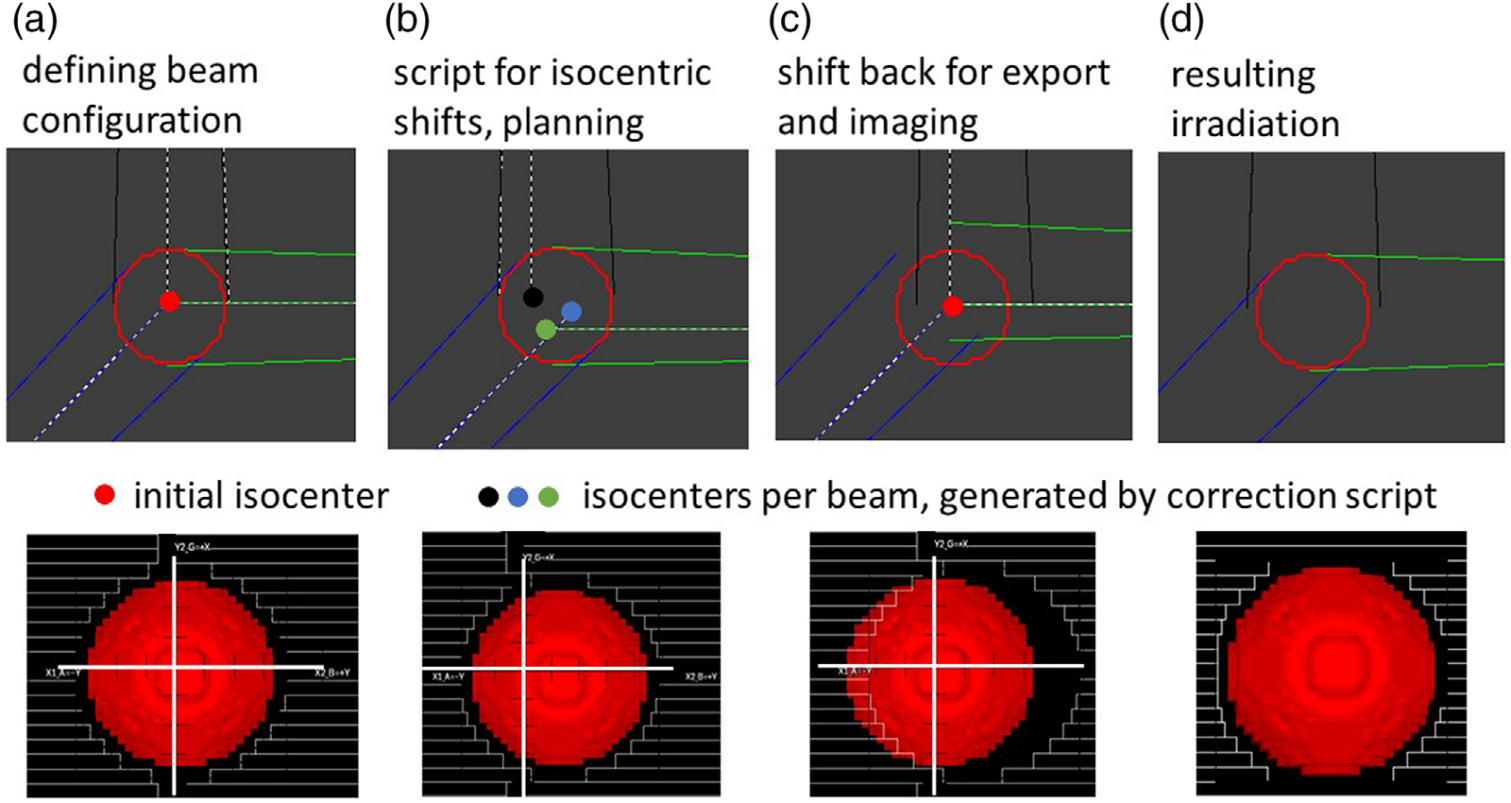
Scheme of the correction procedure. Beam arrangement (lines), isocenters (dots), and PTV shape (contour) displayed in the top row. Field shaped by MLC (white rectangles) relative to the PTV (3D object) displayed in the bottom row. (a) The beam configuration is set. (b) Geometric deviations are accounted for by a script anticipating the isocentric shifts. Apertures are defined at this configuration. (c) The beams are aligned to the ideal isocenter without changing the field shape. This plan is exported. (d) The resulting irradiation will provide target coverage close to what was seen in the treatment planning system at step (b)

The modified protocol presented here is to incorporate these shifts already during the planning process of plans using non‐IMRT fixed beams. The modifications are included prior to the irradiation, as follows (see Figure 1):

One point in the target volume is set as the initial isocenter. The necessary beams are included into the plan. Here, the desired gantry, collimator, and couch angles are already chosen (Figure 1a). Normally, one would continue planning using this configuration. Instead, the script is run, creating multiple new points, one for each angle combination, and setting each beam to its corresponding planning isocenter (Figure 1b). The existing script was altered to contain the updated geometric parameters for both Linacs. Next, planning is carried out as usual and apertures are shaped, for example by using block structures around the target volume or by creating segments by hand until the desired dose distribution is reached. This yields the “true” dose distribution that needs to be reviewed. Upon accepting this plan for treatment it needs to be verified that all MLC positions will remain stationary, that is no blocks are left in the fields, and that the number of monitor units is set to a fixed value. All beams are aligned to the initial isocenter again (Figure 1c). At this stage, the plan is exported to the record and verify system. In the exported plan, the alignment of the field opening to the PTV in the beams eye view will not match and the newly calculated dose distribution may not indicate complete coverage of the PTV. Assuming fairly symmetric PTVs, this visualizes the offset of the uncorrected beam to the target volume. The effect of the corrections on the dose distribution can be estimated by comparing the dose distribution after application of the shifts to the one after moving all beams back to the mean isocenter. Having anticipated and corrected the geometric shifts occurring during the treatment, the resulting irradiation will provide target coverage close to what was initially planned (Figure 1d).

### 2.3 Dosimetric impact and validation measurements

Fifteen recent clinical stereotactic treatments of the brain region were extracted from the database and replanned according to the above‐mentioned procedure keeping the original angles. Thus, for each plan, three sets of beams were produced: One without any changes, referred to as the original plan, and two with changes to account for the geometric deviations observed in Linac 1 and 2, called modification 1 and modification 2, respectively. The test plans included 9–11 beams each. While the typical stereotactic treatment of a single lesion sticks to a combination of ten fixed irradiation directions according to departmental policy, further angular combinations are usually chosen with respect to the PTV position as well as the consideration of overlap when more than one lesion is irradiated. In total, the fifteen test plans included 54 different gantry and couch angle combinations. The collimator angle was typically adjusted to the PTV shape, so different collimator angles were included in the test plans.

The dosimetric impact of the isocentric corrections for the planned treatments was studied by comparing the respective dose volume histograms (DVH). Treatment plans using an ideal, point‐like isocenter and plans with the isocenter shifted by the predicted amount, without readjusting the MLC, were compared. The minimum dose to 98% of the target volume (D98) was determined and serves as an indicator for the near‐mimimum dose.

All three sets were measured using the SRS MapCHECK inserted into the Stereophan for buildup (Sun Nuclear Corporation, Melbourne, FL, USA). Here, we used the SRS MapCHECK, which contains 1013 diodes with an area of 0.48 × 0.48 mm^2^ and diagonally spacing of 2.47 mm center to center distributed on a measurement plane. The system further allows irradiation from any couch and gantry angle combination and, with the Stereophan, provides an outer geometry similar in diameter to a head. We positioned it parallel to the couch. Prior to the measurement, the phantom was aligned to the kV isocenter using CBCT imaging, following the typical procedure for patient treatments and using the clinical head preset with filter settings of F0S20 (like F0S10, but nominal irradiated length at isocenter 276.7 mm). The array was cross‐calibrated using a 10 × 10 cm^2^ 6 MV field with a known dose. As this array allows irradiation from arbitrary angles without the need to spare the electronics, all irradiations were delivered as planned, including the couch angles. The electronics were never in the entry path of the beam in front of the detector array.

The dose distributions of all measured plans were compared to the calculation of the original plan on a virtual phantom using SNC Patient software (Sun Nuclear Corporation, version 8.3) by means of gamma analysis. The typically used gamma pass rate only categorizes the measurement points into pass and fail. The actual gamma value of each measurement point is a better choice to quantify small differences. Therefore, average gamma values including all measurement points were obtained for an absolute global gamma 2%/2 mm with a low dose threshold of 20%. This is an unusually high threshold, but with 9 to 11 fields per plan, this choice avoids detecting small geometric shifts at the beam edges far from the target and restricts the analysis to the more relevant region where dose contributions from different beams overlap. All planned dose distributions were calculated and exported using (1 mm)^3^ voxel size. While Low and Dempsey^15^ suggest using a spatial criterion as three times the calculation grid size or more for accurate calculation of gamma values in steep dose gradient regions, here, for the calculation grid size of 1 mm a stricter criterion of 2%/2 mm was chosen, as in Wack *et al*.^4^ (see this reference for a further discussion of the choice). The common clinical choice of a gamma pass rate was also evaluated for the 1%/1 mm criterion.

## 3 RESULTS

### 3.1 Isocenter corrections

The results of the Winston‐Lutz test are listed in Table 1. Uncertainties based on three measurements with an independent phantom setup are given for Linac 1 and are typically small compared to the magnitude of the included corrections.

**TABLE 1:** Obtained offsets in mm. The MV isocenter is defined as the point with the minimum distance to all three rotation axes (gantry, collimator, couch). The distance between collimator rotation axis and isocenter is evaluated at gantry 0° and applied to all angles. Values 0.5 mm and above are printed in bold. Data in brackets indicates one standard deviation over three consecutive measurements setting up the phantom new each time

Between	Direction	Distance Linac 1	Distance Linac 2
MV isocenter and kV isocenter	left(−)/right(+)	**−0.5** (0.06)	−0.2
	down(−)/up(+)	0.3 (0.06)	0.1
	target(−)/gun(+)	**−0.8** (<0.01)	0.2
Couch rotation axis and kV isocenter	left(−)/right(+)	**0.9 (0.12)**	**0.6**
	target(−)/gun(+)	0.5 **(0.06)**	**0.8**
Collimator rotation axis and MV isocenter		**0.5** (0.06)	0.05
Gantry sag (amplitude)	target(−)/gun(+)	**1.1 (<0.01)**	**1.3**
Gantry wobble (amplitude)	left(−)/right(+)	0.26 (0.11)	0.24
	down(−)/up(+)	0.27 (0.08)	0.26

Linac 1 showed larger deviations than Linac 2 for the distance between kV and MV isocenter. The distance between the collimator and gantry rotation axes was larger for Linac 1, too. The other parameters were similar between the machines.

The sphere containing all beam centers from all studied directions including gantry, collimator, and couch rotation had a diameter of 1.3 mm for Linac 1 and 1.2 mm for Linac 2. Thus, both Linacs are able to achieve a targeting accuracy below 1 mm for all beams as stated as minimum requirements in the AAPM‐RSS Medical Physics Practice Guideline 9.a for SRS‐SBRT.^1^

### 3.2 Dosimetric impact of the corrections

**FIGURE 2:**
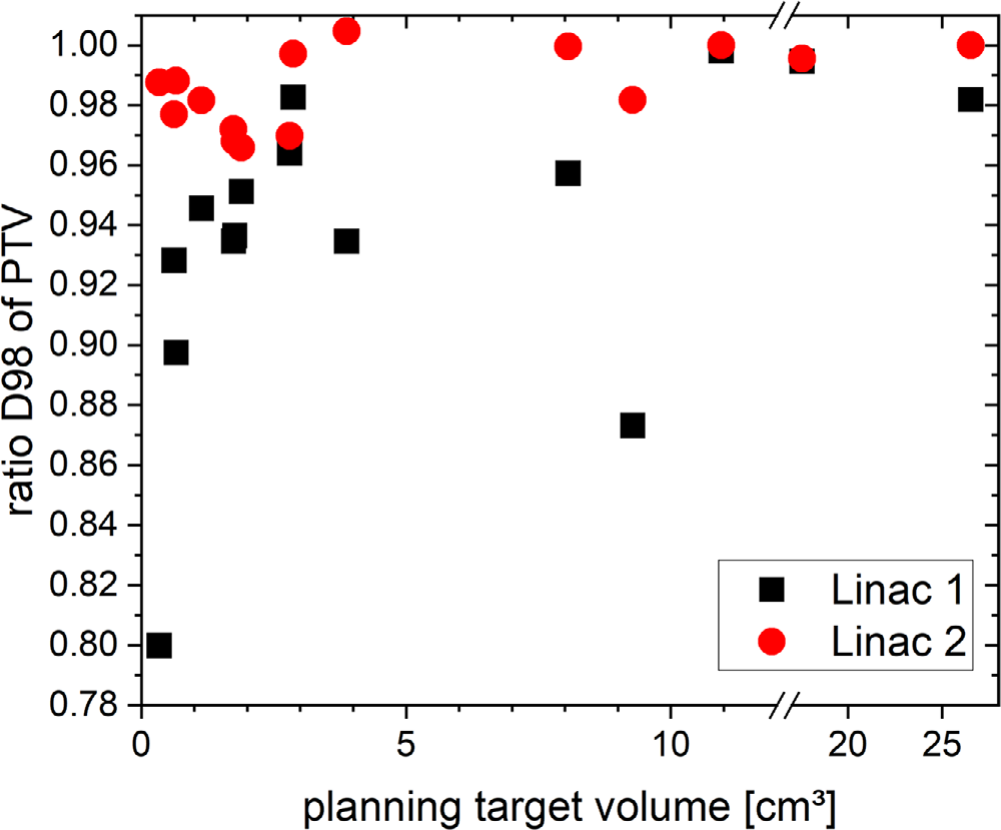
Ratios of the minimal dose to 98% of the target volume (D98) of plans not accounting for isocentric shifts to plan plans accounting for isocentric shifts in the planning system. Beam isocenters shifted according to the geometric deviations found for the respective Linacs

The PTV coverage of the fifteen test plans was sensitive to the application of the isocentric shifts (Figure 2). The reduction of D98 values predicts the deviations expected without the application of the corrections. It generally showed a size dependence, with smaller target volumes usually being prone to larger reductions. D98 values for some of the plans, typically with larger PTVs, were indifferent to the corrections. In one case, D98 coverage even decreased when introducing the shifts yielding a ratio just above 1. Such a situation may arise even though the correction works in general, for example, for highly irregular target volumes and non‐ideal apertures in the initial plan combined with the finite size of the dose grid in combination with submillimeter shifts. Differences between the two Linacs were visible: While the geometric effects for Linac 2 did not exceed a 4% reduction of D98, the dose coverage on Linac 1 was notably lower for most cases with some exceptionally affected plans with a D98 reduced by 20% in the extreme case of a very small PTV.

### 3.3 Validation measurement results

**FIGURE 3:**
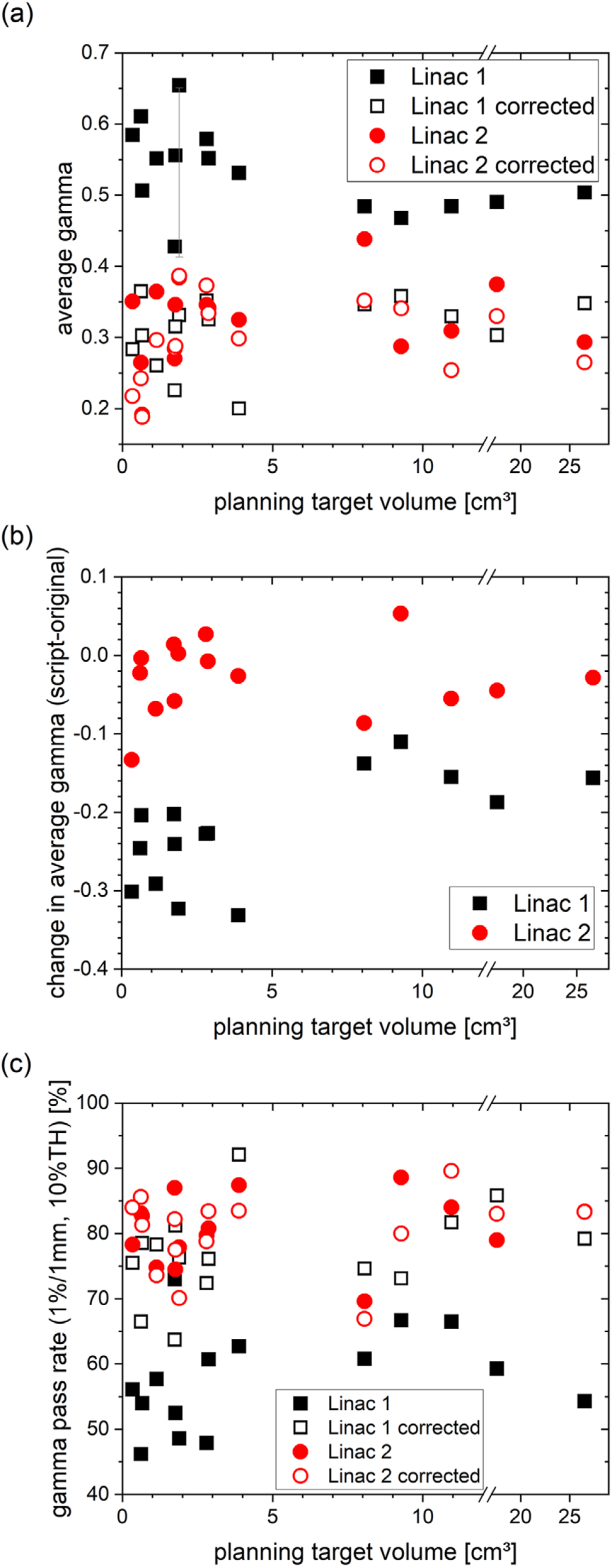
(a) Average global gamma (2%/2 mm, absolute, global) for original plans and plans corrected for geometric deviations by applying isocentric shifts for two different Linacs. Exemplary uncertainties are indicated as two standard deviations from three measurements. (b) Changes in average gamma values between the corrected and the original plan. (c) Gamma pass rates (1%/1 mm, absolute, global, 10% threshold)

The agreement between the calculated dose distribution and the measured dose on the phantom typically improved when the geometric deviations were corrected during the planning process (Figure 3). Geometric corrections for Linac 2 only led to a small improvement of the average gamma (2%/2 mm) from (0.32 ± 0.06) to (0.30 ± 0.06). Applying the corrections to Linac 1 reduced the average gamma from (0.53 ± 0.06) to (0.31 ± 0.05), producing results very similar to Linac 2. The improvement was statistically significant as confirmed by a *t*‐test for connected samples yielding *p* ˂ 0.01 and *p* = 0.03 for Linac 1 and 2, respectively. As there were several plans that already had gamma passing rates of 100% on Linac 2 prior to the correction, the analysis of the average gamma was carried out to show further improvements.

An estimation of uncertainties for the gamma analysis based on three repeated measurements of an exemplary plan each with an independent setup is included (Figure 3a).

## 4 DISCUSSION

Geometric imperfections of the Linac isocenters, rotation axes, and gravitational effects can alter the dose distribution and were typically most pronounced for small targets. Differences between two matched Linacs were clearly observable. A correction of the geometric deviations using a script during the treatment planning process was feasible and improved the agreement between planned and measured dose distributions.

The necessary corrections for the script were retrieved from measurements. All corrections are of the same order as those reported by Wack et al. for the same two Linacs, which were described as stable over time.^4^ However, this should be verified by each user for their machine prior to implementing a correction and be reflected in the additional QA needs coming with the use of such a correction procedure. The newly obtained values (Table 1) are not identical to those reported by Wack et al.^4^ having carried out routine recalibration and a larger repair on Linac 1 affecting the beam steering and having switched to a different protocol using an improved test object for the Winston–Lutz test and in the meantime. The observed gantry sag amplitude of 1.1 to 1.3 mm is comparable to literature data. For nine Elekta Linacs the average sag (in the in‐plane direction) was 1.19 mm.^7^ For 12 Varian accelerators of different models, the sag was within 1 mm for all Linacs.^8^

For most irradiations of small lesions on a non‐perfectly aligned Linac, a decline in coverage compared to the planned dose distribution is expected for geometric reasons. For some cases, only minor changes can be expected. Since dosimetric consequences were generally larger for smaller target volumes, one possible course of action is applying a correction procedure especially to smaller lesions. However, in some cases even the coverage of larger volumes could be considerably improved when applying the script. The PTV shape plays a role here. Therefore, it may be useful to first run the script on a preliminary plan to estimate the dosimetric impact of the geometric deviations for the individual plan. Limiting the application of the correction procedure to only those plans that benefit, reduces additional planning and quality assurance workload.

As a second point, the findings also illustrate that there may be differences between structural identical Linacs. While the consequences of individual offsets are not a priori clear, their inclusion into a comprehensive correction procedure visualizes the dose effects. This helps identify the Linac to be preferred in institutions with multiple available Linacs. In our case, Linac 1 and Linac 2 are each worse than the other in at least one geometric parameter. Ultimately, Linac 1 greatly profits from corrections, while improvements on Linac 2 are marginal. Therefore, the geometrical errors should be quantified for every clinical Linac with high accuracy. Here, it was done by means of the QualiForMed equipment. Frequent measurements showed that the shifts are constant over time. Even for shifts below 1 mm an improvement of the accuracy could be shown. Especially for small targets, the improvement is relevant. Moreover, the application of the correction procedure demonstrates how much individual parameters influence the dose distribution of certain plans, so it becomes clearer where to concentrate efforts for further improvements. In our case, the coincidence of the kV imaging isocenter and the MV isocenter on Linac 1 is such a parameter. A first attempt should be the mechanical correction of systematic offsets on the machine level and the residual effects should then be corrected on the software level. Additionally, it may be necessary to adapt the correction software to the dominating types of Linac geometric imprecisions. For example, the couch wobble correction was small compared to the other corrected geometric parameters and neglected here, but may have to be included for other machines.

The correct irradiation depends on the correct application of the script, which creates a need for additional validation of the planning process. For example, in one case, the script was unintentionally applied to a point other than the planned isocenter. Additional care must be taken that the plan is prepared for the correct Linac keeping in mind any last‐minute changes. Moreover, the beam configuration must be fixed prior to applying the corrections. Any corrections of the beam angle after the script has been run lead to the wrong corrections being applied. Although the details of possible sources of error may vary depending on the treatment planning system and planning workflow, there is an additional risk added by applying the corrections. Quality assurance of individual treatment plans on the exact machine used for treatment becomes necessary. As these checks of treatment plans will become routine, a reliable but quick method needs to be chosen. For the checks to reveal any errors, the setup of the phantom needs to be carried out in the same way as the patient setup, which would typically be a CBCT. Phantom irradiation must be possible from any direction to record also non‐coplanar fields. Further, the used device needs spatial resolution that is high enough to be sensitive to small changes. Over that, voxel sizes below 1 mm would be desirable for dose calculation. Spatial resolutions for calculation and measurement are limited and of the same order as our findings. However, reducing the grid size in the planning system is not meaningful as the planning system was commissioned, thus optimized and validated, at a calculation resolution of 1 mm. Further reduction of the grid size gave a lower average gamma for all test cases. Nevertheless, the resolutions were high enough to demonstrate the effects.

In summary, in addition to implementing the correction script in the TPS regular constancy checks are necessary in the clinic: The obtained isocentric shift values serving as input into the correction script need to be verified at regular intervals, ideally at the same intervals as suggested for constancy checks of geometric parameters for stereotactic radiotherapy, as detailed for example in the AAPM‐RSS Medical Physics Practice Guideline 9.a. for SRS‐SBRT.^1^ The correct application of the script for each treatment plan including the inclusion of the correct Linac needs to be validated, ideally with a measurement on the respective Linac.

Average gamma values comparing the measured with the calculated dose distributions are the basis to judge the accuracy of the irradiation with and without the correction procedure (Figure 3). The indicated uncertainty of the average gamma is given as a mean value of three repeated measurements. The main contributions to the uncertainties were the setup of the measurement device using image‐guidance and geometric reproducibility of the Linac field sizes, gantry, collimator, and couch angles.

An improved agreement of the measured and the calculated dose was demonstrated after carrying out the correction procedure. The validation of the correction script was carried out using a planar instead of a three‐dimensional array, based on the availability of measurement devices with high resolution. All measurements were done with the array‐oriented parallel to the couch top. In principle, the phantom allows any orientation of the measurement plane. While orienting the array with its measurement plane perpendicular to the couch top in a second measurement would provide additional information on the positioning accuracy in the third direction (up‐down), it would also double the measurement time.

Given a detector spacing of 2.47 mm and detector area of 0.48 × 0.48 mm^2^, the filling factor is an issue. The dimensions of the SRS MapCHECK diodes are capable to detect gradient shifts of the order of 1 mm, hence, just in the range of the corrections. Real penumbrae are in the range of 5 mm and distance errors are well detectable. Empirically (Figure 3), we found that the average gamma is very sensitive to the small sub‐millimeter shifts.

**FIGURE 4:**
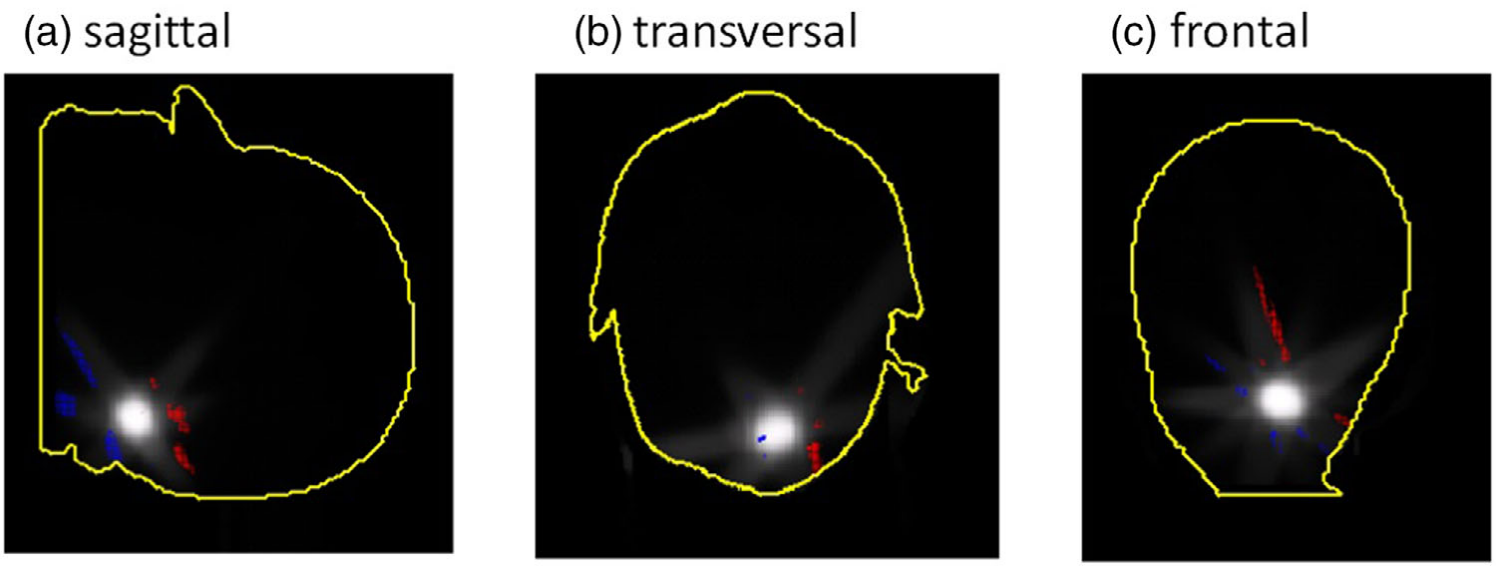
Exemplary gamma analysis (1%/1 mm, no threshold) in the patient anatomy visualized using Sun Nuclear 3DVH software comparing the dose distributions of the original plan with the plan including the isocentric corrections. Points failing the gamma criterion are indicated as red or blue, the dose distribution is indicated in white

Another alternative to check geometric accuracy leaving out dose information are checks using the portal dosimetry system, irradiating each field with a radio‐opaque object in the isocenter and evaluating the field position with respect to the isocenter. This method reaches its limits with many non‐coplanar fields due to possible collisions of the couch with the imager at various positions. Additionally, images of non‐central segments do not contain an isocentrically placed object. Due to the limitation of both methods, a true three‐dimensional phantom would be ideal, for instance, based on radiochromic material or gel. The comparison of three‐dimensional dose distributions in the patient anatomy (Figure 4) illustrates the advantages of such an analysis. Besides, we recommend testing the correct implementation of a correction procedure by correcting and validating the improvement for single fields from gantry and couch combinations including common and extreme angles before moving to clinical plans.

While it was shown for one planning system that applying corrections to stereotactic irradiations leads to higher geometric and, consequently, dosimetric accuracy, and that it is feasible to correct geometric inaccuracies using a script in the Pinnacle planning system, the general idea of the correction procedure can be used ubiquitously, independent of the planning system, irradiation technique, and location. The proposed correction improves the irradiation of stereotactic irradiations for static beam angles, such as fixed fields or IMRT. Especially small lesions treated with stereotactic body radiotherapy profit from the corrections of gantry sag and wobble. The correction procedure is applicable to IMRT plans. Ultimately, it is desirable to implement the corrections also to the individual control points of VMAT plans from any angle, which is currently not possible with the Pinnacle planning system. Alternatively, the necessary modifications of leaf positions may also be done in the digital imaging and communications in medicine (DICOM) RTPLAN files directly instead of using the planning system. Ideally, the Linac control software performs the necessary corrections. Additionally, some changes of the geometry are approximated as isocenter shifts in this correction procedure. For example, the gantry tilt, due to gravitational effects, is described by the gantry flex. An alternative to correct these effects is an adjustment with the rotation of a six‐degree‐of freedom couch, which brings technical challenges as it requires synchronization of the correction script with the couch. However, additional benefits can be expected for irradiation of multiple targets using a single isocenter.

## 5 CONCLUSIONS

Geometric deviations of the isocenter can lead to reduced dose coverage when irradiated on standard C‐arm Linacs compared to the visualization of the dose distribution in the TPS. The magnitude of the effect depends on both the PTV size and shape and the Linac's geometric accuracy. It was shown that a correction of the isocentric shifts during the initial planning process and the dosimetric validation is feasible. Following this procedure leads to better agreement between measured and calculated dose distributions than for the uncorrected plans. Consequently, the accuracy of the delivered dose distribution to a patient could be improved. As the necessary corrections vary even for Linacs of the same type, it should be evaluated which of the available Linacs and which plans profit from a correction. Necessary quality assurance of the plans after manipulations with the script can be performed with available commercial measurement phantoms although a phantom measuring in true 3D would be desirable.

## AUTHOR CONTRIBUTIONS

S.W., R.S., and O.S. designed the study and critically discussed the results. R.S. and S.W. prepared and carried out the experiments and analyzed the data. S.W. wrote the manuscript with input from all authors.

## CONFLICT OF INTEREST

The authors declare no conflict of interest.
